# Association between primary aldosteronism and type 2 diabetes and identification of potential molecular mechanisms

**DOI:** 10.3389/fendo.2026.1863900

**Published:** 2026-08-04

**Authors:** Chaoyan Tang, Liheng Meng, Haiyun Lan, Jing Xian, Cuihong Chen, Lijun Pang, Yingfen Qin

**Affiliations:** 1 Department of Endocrinology, The First Affiliated Hospital of Guangxi Medical University, Nanning, Guangxi, China; 2 Department of Endocrinology, The Six Affiliated Hospital of Guangxi Medical University, Yulin, Guangxi, China; 3 College of Pharmacy, Guangxi Medical University, Nanning, China

**Keywords:** AGE-RAGE pathway, bioinformatics analysis, mendelian randomization, primary aldosteronism, SRC, type 2 diabetes

## Abstract

**Background:**

Primary aldosteronism (PA) is a common cause of secondary hypertension and is associated with an increased risk of cardiovascular and metabolic complications. Emerging evidence suggests a close relationship between PA and type 2 diabetes mellitus (T2DM), with a higher prevalence of impaired glucose metabolism observed in PA patients. However, the underlying molecular mechanisms linking PA and T2DM remain unclear.

**Purpose:**

This study aimed to investigate the causal relationship between PA and T2DM and to explore the underlying molecular mechanisms and potential therapeutic targets.

**Materials and methods:**

Mendelian randomization (MR) was performed to evaluate the causal association between PA and T2DM. Shared disease-associated genes were identified from public databases, followed by protein-protein interaction (PPI) construction and pathway enrichment analysis. Experimental validation was conducted in NCI-H295R and HepG2 cells. Virtual screening and molecular docking were applied to identify candidate small-molecule compounds targeting key genes.

**Results:**

MR suggested a potential positive causal association between PA and T2DM. Integrated bioinformatics and experimental analyses identified SRC as a central regulatory hub gene in PA-associated metabolic dysfunction. Functional experiments showed that aldosterone-induced SRC activation impaired insulin signaling through the AKT-IRS1-GLUT4 axis, while SRC inhibition partially restored insulin response. Pathway analysis highlighted the AGE-RAGE signaling pathway as a key inflammatory mediator linking PA to T2DM. Furthermore, SRC-mediated metabolic signaling and AGE-RAGE-driven inflammatory responses appeared to synergistically contribute to disease progression. Virtual screening identified Grossularine-1 as a promising SRC-targeting compound.

**Conclusions:**

Our findings demonstrate a genetic association between PA and T2DM, with SRC identified as a potential key mediator in their pathogenesis. We further highlight the pivotal role of the AGE-RAGE pathway in PA-induced T2DM and propose Grossularine 1 as a promising candidate.

## Graphical Abstract

**Figure:**
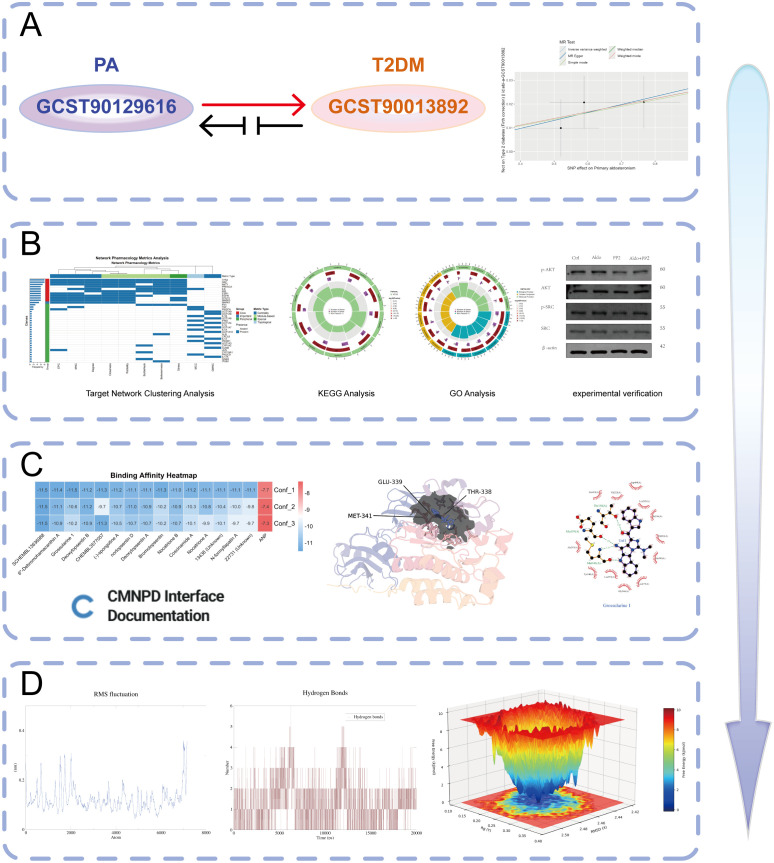
Workflow for exploring the mechanism of action of PA on T2DM and predicting active molecules on the basis of Mendelian randomization and virtual screening. **(A)** A Mendelian randomization analysis revealed a significant positive association between PA and T2DM. **(B)** Core targets were identified by bioinformatics analysis of comorbidity genes and then validated experimentally. **(C)** Active molecules were predicted via virtual screening. **(D)** Molecular dynamics simulation of Grossularine-1 and SRC.

## 1 Introduction

Primary aldosteronism (PA) usually refers to the autonomous secretion of aldosterone by the adrenal cortex, leading to water and sodium retention in the body, increased blood volume, and inhibition of the renin-angiotensin system activity (1). The main clinical manifestations are hypertension and hypokalemia, but only 8% of the PA patients will have both conditions simultaneously. Most PA cases are sporadic, with approximately 5% of PA patients being familial (2). This excessive aldosterone production also increases the excretion of potassium and hydrogen in urine, resulting in hypertension, hypokalemia, and metabolic alkalosis (3). There is a close relationship between aldosterone and hypertension, and PA is often detected in the systematic screening of hypertensive patients (4). Several reports consistently indicate that the prevalence of PA in hypertension ranges from 5% to 10%. However, with the advancement of research and detection methods, it is now believed that the prevalence may be as high as 20% (5). In a meta-analysis including PA and essential hypertension (EH) patients, the risk of cardiovascular events and target organ damage in PA patients was significantly higher, including stroke, coronary artery disease, heart failure, type 2 diabetes (T2DM), and the metabolic syndrome (2).

Among these comorbidities, T2DM is of particular clinical importance due to its global burden and metabolic interaction with hypertension. Approximately 537 million adults worldwide have T2DM, accounting for 10.5% of the adult population (6). Global diabetes-related medical expenditures amount to 966 billion US dollars, accounting for 11% of global health expenditures (7). The association between PA and T2DM was first described over 40 years ago in patients with PA and impaired glucose tolerance (8). Studies have shown that the incidence of T2DM and metabolic syndrome in PA patients is significantly higher than that in healthy controls, and 35% of non-diabetic PA patients have abnormal oral glucose tolerance test (OGTT), with significant improvement in blood glucose after PA treatment (9). Additionally, studies have also shown that the incidence of PA in the diabetic population is significantly higher than that in the general population (10, 11). Regardless, there may be some complex connections between the occurrence and development of PA and T2DM. However, the specific molecular mechanisms underlying their occurrence remain unclear.

Studies have shown that aldosterone can block insulin-induced glucose uptake and induce the degradation of IRS-1 and 2, an effect that depends on the generation of reactive oxygen species (12). In human adipocytes, aldosterone also impairs insulin sensitivity, indicating that aldosterone can cause insulin resistance in adipose and vascular tissues. Moreover, aldosterone negatively affects the structural and functional integrity of pancreatic β cells by promoting inflammatory and oxidative stress conditions, thereby reducing insulin release and action (including in blood vessels) (13, 14). These findings suggest that aldosterone excess may contribute to the development of T2DM; however, the underlying molecular mechanisms remain incompletely understood, particularly at the gene regulatory and pathway levels. In this study, MR was applied to assess the genetic relationship between PA and T2DM, and then used disease co-gene target analysis to identify common genes and molecular pathways mediating the occurrence of the two diseases. Furthermore, we have verified through experiments that SRC plays a crucial role in PA-mediated T2DM. Finally, we used network pharmacology to find small molecule drugs that may treat PA-mediated T2DM. Through our research, we aim to provide new molecular mechanisms and therapeutic drugs for PA patients to prevent and treat the occurrence of T2DM.

## 2 Methods

### 2.1 Mendelian randomization

A two-sample MR design was employed to examine the potential genetic association between PA and T2DM. Independent single-nucleotide polymorphisms (SNPs) identified from genome-wide association studies (GWASs) were selected as instrumental variables (IVs). In accordance with MR analytical requirements, exposure and outcome datasets were obtained from the IEU Open GWAS Project database (https://gwas.mrcieu.ac.uk). The genetic phenotype “PA” was retrieved from the IEU Open GWAS database, yielding the PA dataset (GCST90129616). This dataset pertains to the European population and comprises 978 PA individuals. In addition, the T2DM dataset (GCST90013892) was included. This dataset is also for the European population and includes a total of 406, 831 T2DM individuals. SNPs with genome-wide significance (*P *< 5 × 10^-6^) were selected, and allele orientations were manually harmonized using the dbSNP database. Linkage disequilibrium (LD) clumping was performed using the European population reference panel (window size = 5, 000 kb, r² < 0.01), resulting in three independent genetic instruments. Heterogeneity was assessed using Cochran’s Q test, while horizontal pleiotropy was evaluated via the MR-Egger intercept test. Leave-one-out analysis was conducted to determine the influence of individual SNPs, and MR-PRESSO was applied to detect potential outliers. More detailed MR analytical procedures can be found in our previous study (15).

### 2.2 Acquisition of disease target genes and construction of the protein-protein interaction network

To comprehensively analyze the underlying mechanisms linking PA and T2DM, a disease target gene analysis approach was applied. Disease-associated target genes were retrieved from public databases, including GeneCards, CTDbase, and TTD. After removing duplicates, overlapping genes between the two disease datasets were identified and used for subsequent protein-protein interaction (PPI) analysis. The PPI network was constructed using the STRING database and visualized in Cytoscape. Hub genes were identified based on multiple topological features using the CytoHubba plugin. Among the candidate genes, SRC consistently ranked as a key hub across different centrality measures, indicating its potential central regulatory role in PA-mediated T2DM.

### 2.3 Pathway enrichment analysis

Core target genes derived from the PPI network were subjected to Gene Ontology (GO) and Kyoto Encyclopedia of Genes and Genomes (KEGG) enrichment analyses using the Homo sapiens background. GO analysis included biological process, cellular component, and molecular function categories, while KEGG analysis was performed to identify significantly enriched signaling pathways associated with the candidate genes.All analyses were conducted using standard bioinformatics tools, and detailed procedures have been previously described (16).

### 2.4 Cell culture; quantitative real-time qPCR and western blotting

The NCI-H295R cell line used in this study was obtained from Procell Life Science & Technology Co., Ltd. (Wuhan, China; Cat. No. CL-0399). The HepG2 cell line used in this study was obtained from Wuhan Zishan Biotechnology Co., Ltd. (Wuhan, China; Cat. No. STCC10114P). The cell lines present in this study were obtained from a commercial supplier and did not require ethical approval. NCI-H295R and HepG2 cells were used as the *in vitro* model system (17). Cells were inoculated into 6-well or 12-well culture plates in DMEM/F12 (high glucose) supplemented with 10% FBS and 100 IU/ml penicillin/streptomycin at 37 °C and 5% CO_2_. Aldosterone was purchased from Millipore and stored at 4 °C (product code: A833832). According to the manufacturer’s instructions, aldosterone was dissolved in DMSO at a concentration of 1 μM and applied to the cells. According to previous studies, a concentration of 1 μM aldosterone can be defined as a high aldosterone level. PP2 was purchased in powder form from MedChem Express (MCE) and stored at 4 °C (product code: HY-13805). PP2 was dissolved in DMSO at a concentration of 10μM according to the manufacturer’s instructions and applied to cells.

The DMSO concentrations in both the control group and the treatment group were the same, and were both less than 0.1%. The next experiment was conducted 24 hours after the administration of aldosterone and PP2. While the aldosterone + PP2 group added PP2 one hour after the administration of aldosterone, and then waited for another 24 hours before conducting the subsequent experiments. The specific operation procedures for Western blotting and quantitative real-time qPCR can be found in our previous publications (16). The RT–qPCR sequences of primers and these antibodies used are shown in **Supplementary Tables 1**, **2**. The quantification of phosphorylated proteins was carried out by calculating the ratio of phosphorylated protein to total protein, and then dividing it by the reference protein for calculation and normalization. Measurements of western blotting strips were performed using Image J 1.52 software.

### 2.5 Virtual screening

Virtual screening was performed using AutoDock Vina to identify potential bioactive compounds from the Comprehensive Marine Natural Products Database (CMNPD) (18) A two-stage docking strategy was applied. In the initial screening, approximately 40, 000 compounds were evaluated to identify candidates with favorable binding affinities. Compounds with binding energies below −11.0 kcal/mol were subjected to a secondary, more rigorous docking analysis using increased sampling parameters and ANP as a reference ligand.Detailed methodological parameters have been previously described (15). Detailed methodological parameters are provided in the **Supplementary Materials**.

### 2.6 Molecular dynamics simulation

Molecular dynamics simulation (MDS) was performed using GROMACS 2020.06 to evaluate the stability of the SRC-Grossularine-1 complex under near-physiological conditions. After system preparation, energy minimization, and equilibration, a 20 ns production simulation was conducted. Trajectory stability was assessed using root-mean-square deviation (RMSD), root-mean-square fluctuation (RMSF), and radius of gyration (Rg) analyses. Further details are available in the **Supplementary Materials**.

## 3 Results

### 3.1 Causal effect of PA on T2DM risk

**Figure 1:**
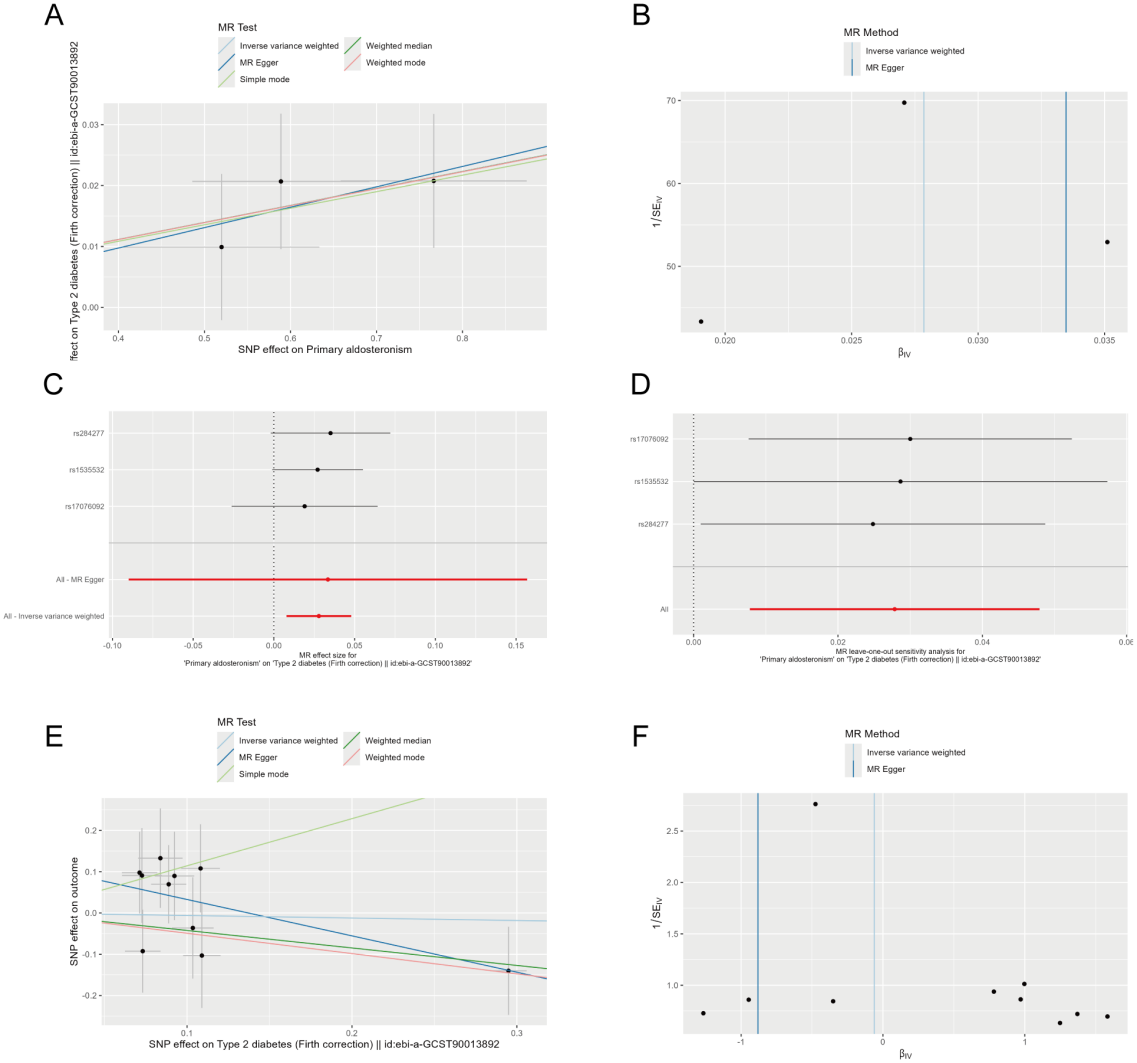
Mendelian randomization analysis of PA and T2DM. **(A, E)** res plot; **(B, F)** funnel plot; **(C)** forest plot; **(D)** leave-one-out plot.

When PA was considered as the exposure and T2DM as the outcome, inverse variance–weighted (IVW) analysis revealed a significant positive association between the two (*p* = 0.0065), indicating that each genetically predicted 1-unit increase in log(OR) for PA was associated with a 2.8% higher risk of T2DM (OR = 1.028, 95% CI: 1.008–1.049) (**Figures 1A–D**). The weighted median method confirmed the robustness of this association (*p* = 0.017). Although the MR-Egger regression did not reach statistical significance (*p* = 0.689), the direction of effect was consistent with the main result; both the weighted mode (*p* = 0.183) and simple mode (*p* = 0.190) also showed a positive trend. Heterogeneity testing suggested good homogeneity of the instrumental variables (*p* = 0.95), and the MR-Egger intercept indicated no evidence of horizontal pleiotropy (*p* = 0.689). Leave-one-out analysis confirmed that no single SNP drove the overall association. Collectively, these sensitivity analyses support the reliability of the findings (**Supplementary Table 3**). However, when T2DM was considered as the exposure and PA as the outcome, inverse variance-weighted (IVW) analysis revealed no statistically significant association (OR = 0.94, 95% CI: 0.55–1.6; *p* = 0.822) (**Figures 1E, F**) (**Supplementary Table 4**).

### 3.2 SRC as potential key gene in PA-mediated T2DM

**Figure 2:**
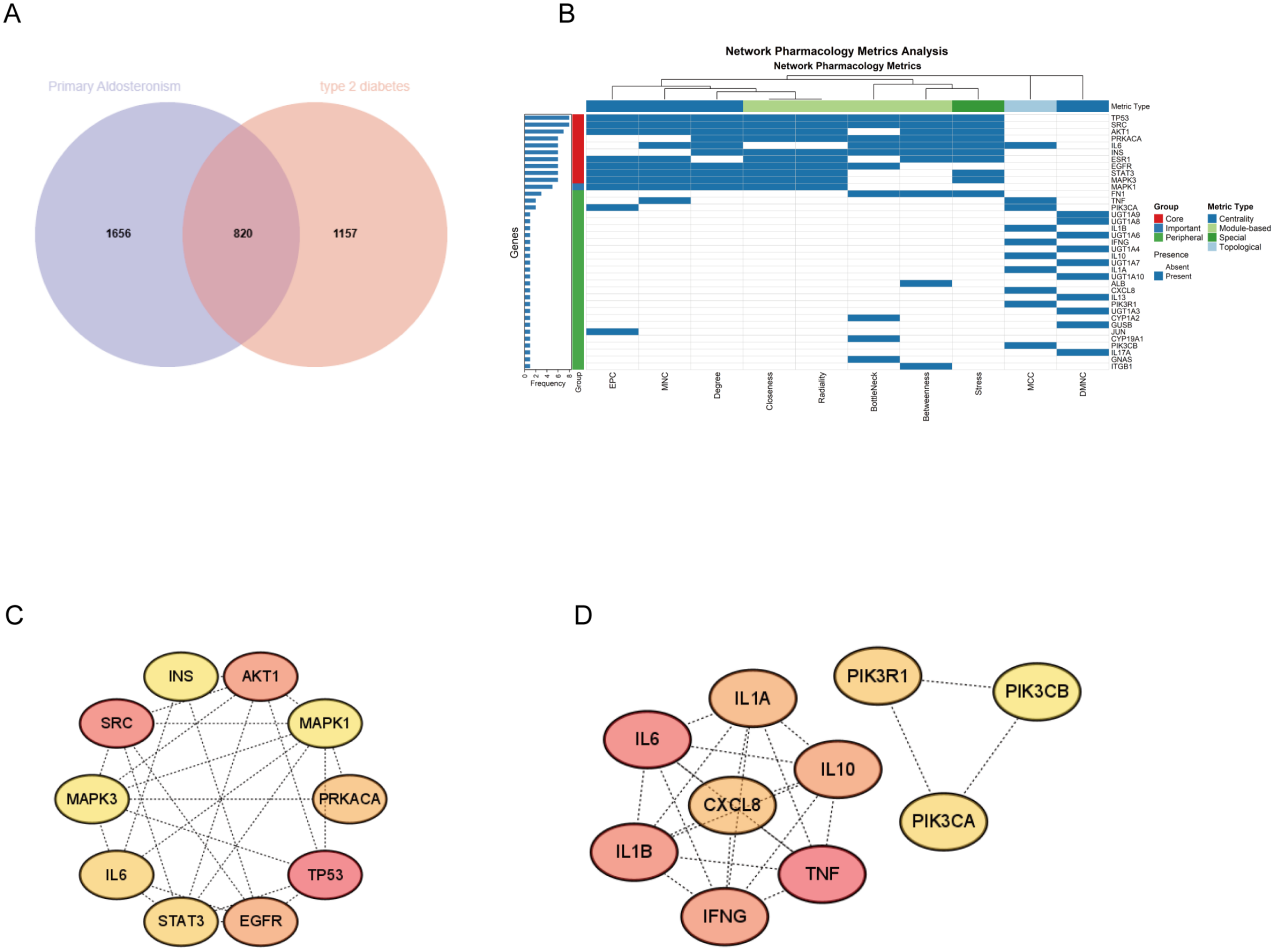
Network characteristics and core gene analysis of overlapping targets between PA and T2DM. **(A)** Overlapping targets of PA and T2DM; **(B)** This heatmap shows the presence (blue) and absence (white) of the top 10 genes in each of the 10 network metrics. The left annotations display the occurrence frequency of genes across metrics and their classification into Core, Important, or Peripheral groups, while the top annotations indicate metric types. Combined with clustering dendrograms, it intuitively presents the distribution characteristics, importance classification of these genes, and the classification features of the metrics; **(C)** Gene network of the top 10 genes ranked by degree value; **(D)** Gene network of the top 10 genes ranked by MCC value.

Disease-associated target genes for PA and T2DM were retrieved from multiple public databases, yielding 2, 476 and 1, 977 genes, respectively, with 820 overlapping targets identified for subsequent analysis (**Figure 2A**). These shared genes were subjected to PPI network analysis, and SRC was identified as a key hub gene based on multiple topological features, consistently ranking among the top candidates across different centrality measures (**Figures 2B–D**). Given its stable and prominent network position, SRC was selected as the primary candidate for further experimental validation and small-molecule screening.

### 3.3 Experimental verification of SRC and AKT1 as key genes mediating PA-mediated T2DM

**Figure 3:**
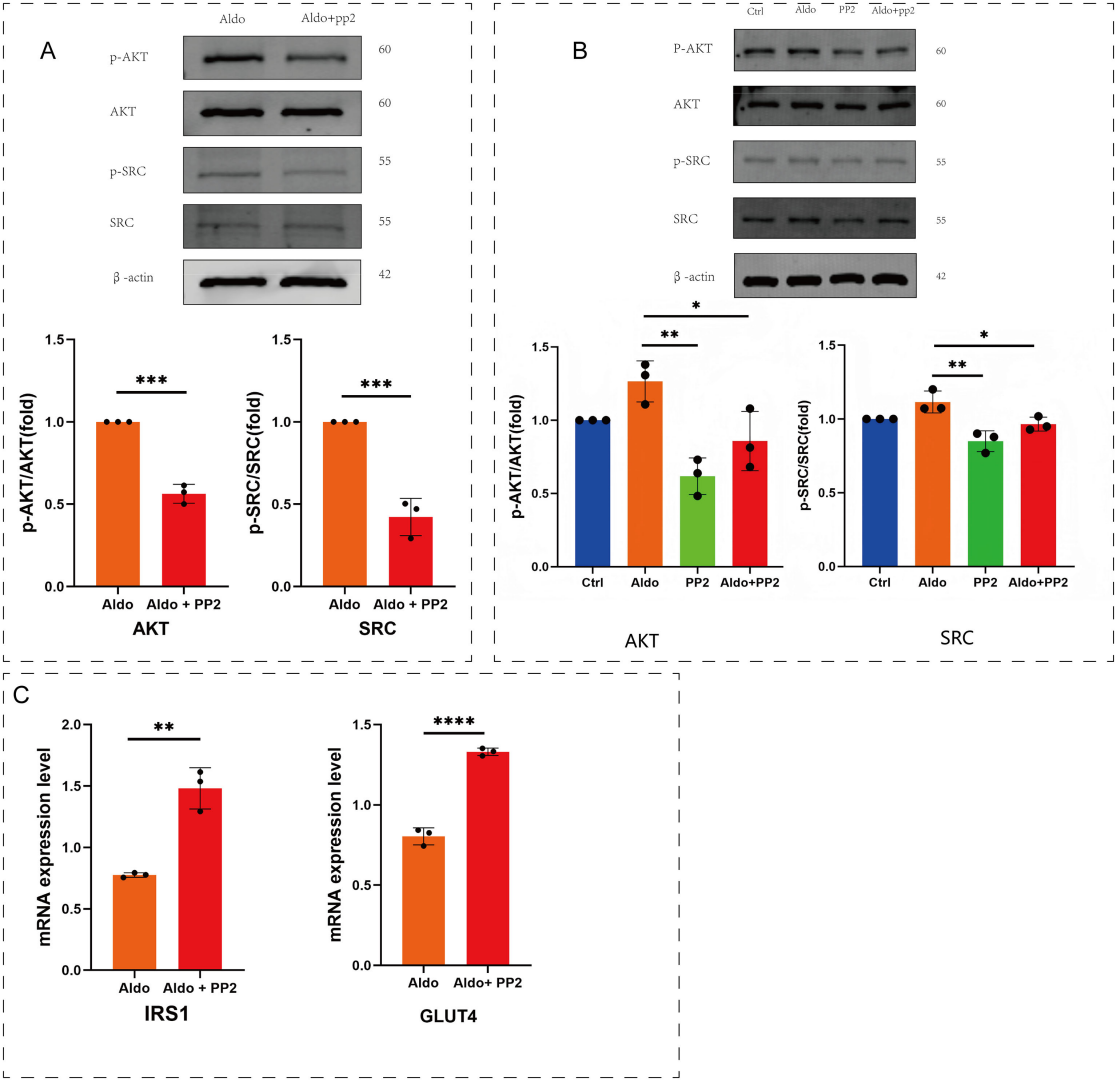
Effects of Src inhibition on molecular expression profiles in NCI-H295R and HepG2 cells. **(A)** Representative Western blot images and quantitative analysis of protein expression in NCI-H295R cells treated with or without the Src inhibitor PP2. **(B)** Representative Western blot images and quantitative analysis of p-Akt/Akt and p-Src/Src ratios in HepG2 cells treated with aldosterone (Aldo) in the presence or absence of PP2. Cells were divided into four groups: Ctrl, Aldo, PP2, and Aldo+PP2. **(C)** Relative mRNA expression levels of IRS1 and GLUT4 measured by quantitative PCR. Data are presented as mean ± SD. Statistical significance was determined between groups (*p < 0.05, **p < 0.01, ***p < 0.001, ****p < 0.0001).

SRC-mediated signaling was further validated in NCI-H295R and HepG2 cells under a high-aldosterone condition. In NCI-H295R cells, Western blot analysis showed elevated phosphorylation of SRC and AKT1 upon aldosterone stimulation, which was attenuated by PP2 treatment (**Figure 3A**). In HepG2 cells, aldosterone similarly induced SRC and AKT1 phosphorylation across four treatment groups, and PP2 significantly reduced their phosphorylation levels (**Figure 3B**). Meanwhile, key insulin signaling components, including IRS1 and GLUT4, were downregulated under high-aldosterone stimulation, whereas SRC inhibition partially restored their expression levels (**Figure 3C**). These findings suggest that SRC activation contributes to insulin signaling impairment in a high-aldosterone environment, supporting its potential role in mediating metabolic dysfunction associated with primary aldosteronism.

### 3.4 AGE-RAGE pathway as a key mediator in PA-induced T2DM

**Figure 4:**
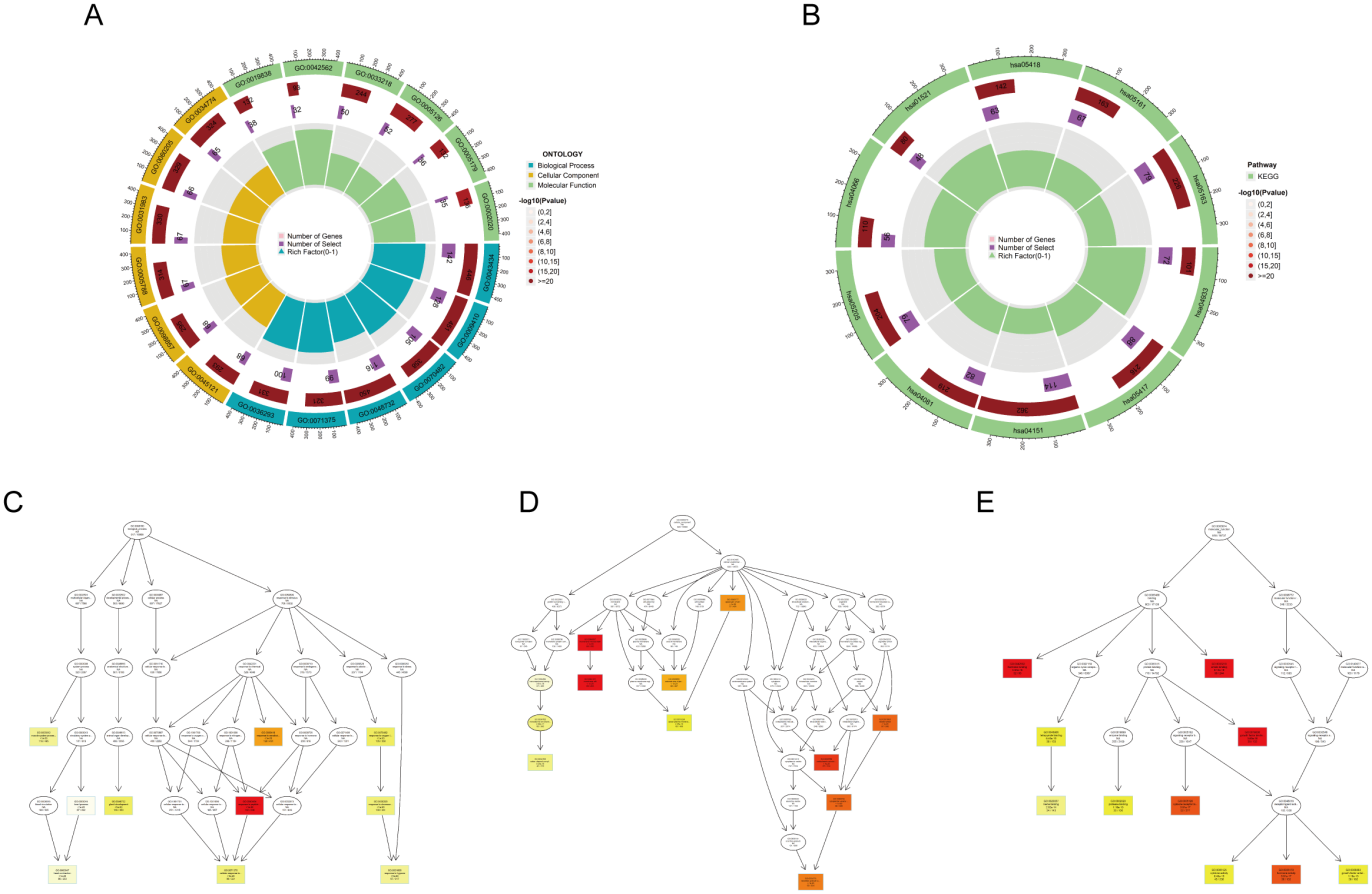
Circular plot visualization of GO and KEGG enrichment analyses for PA and T2DM comorbidity genes. **(A)** This GO circular plot displays the top 6 significant terms in each of the three categories (BP, CC, MF) filtered by adjusted P-values through circular tracks. The inner track labels GO IDs; the outer track adjacent to it shows the number of background genes and their values with a red color scale corresponding to -log10 (adjusted P-value); the next outer purple track presents the number of significant genes and their values; the outermost track uses the height of rectangles in category-specific colors to reflect the enrichment factor. **(B)** The KEGG circular plot illustrates the top 10 pathways via circular tracks, including pathway IDs, number of background genes (with a red color scale corresponding to -log10 (adjusted P-value)), number of significant genes (purple), and enrichment factors. **(C)** GO BP functional network diagram shows gene set’s biological process enrichment relationships. **(D)** GO CC functional network diagram presents cellular component functional connections of the gene set. **(E)** GO MF functional network diagram displays molecular function enrichment patterns and associations of the gene set.

GO enrichment analysis revealed that PA-related T2DM candidate genes were mainly involved in hormonal responses, electrolyte regulation, and nutrient metabolism (BP), and were enriched in membrane-related structures and receptor-associated functions (CC and MF) (**Figures 4A, C–E**). KEGG pathway enrichment revealed significant involvement of differentially expressed genes in the AGE-RAGE signaling pathway, lipid and atherosclerosis pathways, and the PI3K-Akt signaling pathway (**Figure 4B**). Among these, the AGE-RAGE signaling pathway was identified as the key mechanism linking PA to T2DM, potentially through regulation of insulin resistance and metabolic inflammation.

### 3.5 Virtual screening identifies Grossularine-1 as a lead candidate

**Figure 5:**
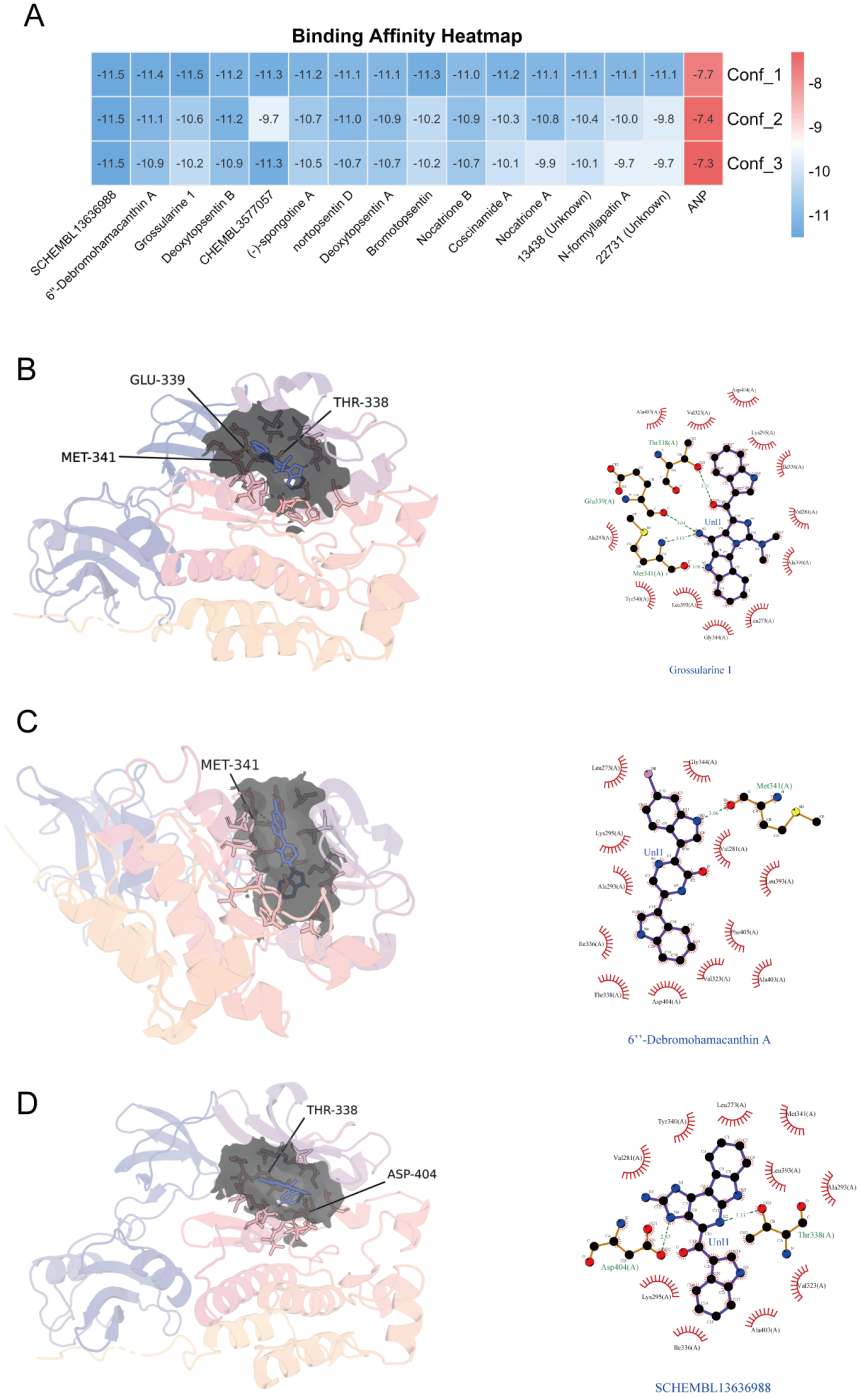
Comparison of binding energies of virtual screening-derived small molecules with SRC and analysis of key conformations. **(A)** Comparison of binding energies between the top 10 small molecules (after secondary screening) and the ligand ANP in SRC; **(B)** Spatial conformation of Grossularine-1 with SRC; **(C)** Spatial conformation of 6’’-Debromohamacanthin A with SRC; **(D)** Spatial conformation of SCHEMBL13636988 with SRC.

Virtual screening identified 15 compounds with binding energies ≤ −11.0 kcal/mol in the initial screening. Among these, Grossularine-1, SCHEMBL13636988, and Bromotopsentin exhibited the highest binding affinities. In the refined docking analysis using ANP as a reference ligand, all candidates showed improved binding performance compared with the reference compound. Considering binding affinity and binding interactions, Grossularine-1 was selected as the most promising lead compound for further molecular dynamics analysis (**Figure 5**).

### 3.6 Molecular dynamics simulations demonstrate stable binding

**Figure 6:**
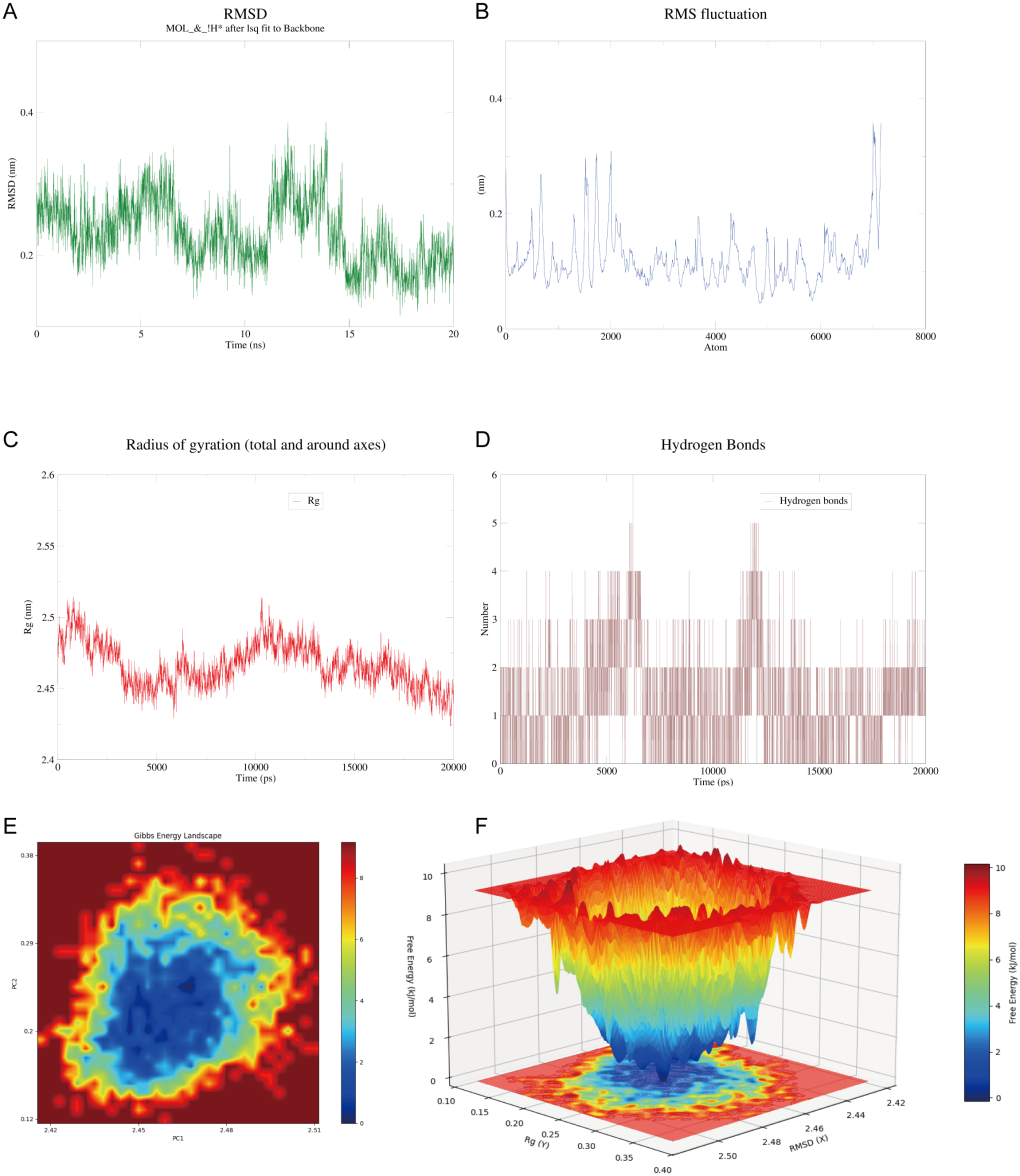
Molecular dynamics simulation of Grossularine-1 and SRC. **(A)** The RMSD curve of SRC with Grossularine-1. **(B)** The RMSF curve of SRC with Grossularine-1. **(C)** The Rg curve of SRC with Grossularine-1. **(D)** The fluctuation curve of the number of hydrogen bonds betweenSRC and Grossularine-1. **(E, F)** Free energy profiles of target SRC with Grossularine-1.

Molecular dynamics simulation showed that the SRC-Grossularine-1 complex remained stable throughout the 20 ns simulation. The RMSD values stabilized around 0.3 nm, indicating a stable binding conformation (**Figure 6A**). RMSF analysis showed limited fluctuations in most amino acid residues, suggesting overall structural stability of the protein-ligand complex (**Figure 6B**). The radius of gyration (Rg) remained stable at approximately 2.0 nm, indicating a compact protein structure during simulation (**Figure 6C**). Hydrogen bond analysis revealed persistent interactions between SRC and Grossularine-1, with an average of two hydrogen bonds throughout the simulation period.

## 4 Discussion

PA is recognized as a common and clinically significant cause of secondary hypertension, leading to more frequent and severe complications such as stroke, atrial fibrillation, hypertensive heart disease, and nephropathy. To date, PA has been identified in nearly 20% of hypertensive patients (19, 20) Compared with essential hypertension, patients with PA exhibit a higher prevalence of impaired fasting glucose and broader glucose metabolism abnormalities, including components of metabolic syndrome (21). Several clinical studies have further demonstrated that PA patients show impaired glucose tolerance and insulin resistance compared with normotensive controls (22, 23). Importantly, Mendelian randomization analysis in the present study provides causal evidence supporting a positive association between genetically predicted PA and increased risk of T2DM, strengthening previous observational findings.

Recent mechanistic studies have explored the relationship between PA and T2DM, with findings suggesting that elevated aldosterone promotes oxidative stress, endothelial dysfunction, and inflammatory activation through multiple pathways (24, 25). These processes collectively contribute to vascular injury and metabolic inflammation. Similar pathological features are also observed in diabetes, indicating shared mechanistic alterations between PA and T2DM (26). Furthermore, aldosterone has been shown to induce insulin resistance and endothelial dysfunction by impairing insulin-stimulated glucose uptake and promoting degradation of IRS-1 and IRS-2 in adipocytes (27, 28). In addition, aldosterone disrupts pancreatic β-cell function through oxidative stress and inflammatory injury, leading to reduced insulin secretion and systemic glucose dysregulation (13, 29, 30). However, despite these findings, the key molecular mediators linking aldosterone excess to T2DM development have remained incompletely defined.

SRC was identified as a central regulatory hub in PA-associated metabolic dysfunction based on network-level analysis. In NCI-H295R and HepG2 cells under aldosterone excess, SRC activation was associated with increased phosphorylation of SRC and AKT, accompanied by suppression of IRS1 and GLUT4. SRC inhibition by PP2 attenuated these effects and partially restored insulin signaling components. These results suggest that SRC acts upstream of the AKT IRS1 GLUT4 axis and mediates aldosterone-induced insulin resistance. Previous studies suggested that this might manifest at two levels: one is as a SRC directly mediating aldosterone-induced peripheral insulin resistance; the other is as a transcriptional co-activator (SRC-1/SRC-2) regulating central energy metabolism and glucose sensing. These two pathways may work synergistically in patients with PA (31, 32).

In addition to SRC signaling, pathway-level analysis revealed AGE-RAGE signaling as another key axis. We hypothesize that the binding of AGEs to RAGE activates NF-κB and ROS pathways, triggering inflammatory responses, β-cell damage, and insulin resistance. Aldosterone may further enhance RAGE expression, establishing a feed-forward loop that exacerbates metabolic inflammation and contributes to T2DM progression (33). Together, these findings suggest that SRC-mediated insulin signaling and AGE-RAGE driven inflammatory pathways may form a coordinated regulatory axis that cooperatively amplifies metabolic dysfunction in PA. Notably, clinical observations have shown that treatment with spironolactone is associated with increased C-peptide levels, further supporting a role of aldosterone in impairing insulin secretion (34). Finally, virtual screening and molecular dynamics simulation identified Grossularine-1 as a potential lead compound targeting SRC, providing a possible therapeutic strategy for PA-associated metabolic dysfunction (35).

This study initially elucidates the relationship between PA and T2DM and identifies the potential mechanism associated with the AGE-RAGE signaling pathway. These findings were further validated through patient samples and clinical data. However, there are some limitations in this study (1): The molecular mechanisms by which the AGE-RAGE signaling pathway and PA influence T2DM have not been fully elucidated (2). The efficacy of the small molecules identified in this study has yet to be further validated (3). The study did not conduct verification of the research results in an *in vivo* model, and the experimental conditions *in vitro* still had deficiencies. Therefore, in future research, we plan to explore the precise molecular mechanisms through *in vitro* and *in vivo* models to investigate how the AGE-RAGE signaling pathway influences the development of both diseases and to validate the therapeutic efficacy of the identified small molecules.

## 5 Conclusions

This study demonstrates a significant association between primary aldosteronism and type 2 diabetes mellitus through Mendelian randomization and integrative bioinformatics analysis. SRC and the AGE-RAGE signaling pathway were identified as key regulatory components involved in PA-associated metabolic dysfunction. Experimental validation further confirmed that aldosterone-related signaling contributes to impaired insulin response. In addition, Grossularine-1 was identified as a potential SRC-targeting compound, providing a candidate for further therapeutic investigation in PA-related metabolic disorders.

## Data Availability

The datasets presented in this study can be found in online repositories. The names of the repository/repositories and accession number(s) can be found in the article/**Supplementary Material**.

## Supplementary material

Supplementary Table 1 The primer pair sequences used for RT-qPCR reactions.

Supplementary Table 2 Key antibody information.

Supplementary Table 3 SNPS in the dataset used for MR analysis.

Supplementary Table 4 SNPS in the dataset used for MR analysis.
