# Supplementary material for: Association between primary aldosteronism and type 2 diabetes and identification of potential molecular mechanisms
Source: Front Endocrinol (Lausanne). 2026 Aug 4;17:1863900. doi: 10.3389/fendo.2026.1863900 (PMC13481238; doi:10.3389/fendo.2026.1863900)
Supplement: Supplementary file 1 [file Table1.docx]

**Table S1** The primer pair sequences used for RT-qPCR reactions

| **Gene** | **sequence** |
| --- | --- |
| GLUT4-F | ACCAGCATCTTCGAGACAGC |
| GLUT4-R | TCCACCAACAACACCGAGAC |
| IRS1-F | CAGTTTCCAGAAGCAGCCAG |
| IRS1-R | GAAGAGGATTTGCTGAGGTCAT |
| β-actin-F | TCACCATGGATGATGATATCGC |
| β-actin-R | CCACATAGGAATCCTTCTGACC |
