# Supplementary material for: Association between primary aldosteronism and type 2 diabetes and identification of potential molecular mechanisms
Source: Front Endocrinol (Lausanne). 2026 Aug 4;17:1863900. doi: 10.3389/fendo.2026.1863900 (PMC13481238; doi:10.3389/fendo.2026.1863900)
Supplement: Supplementary file 2 [file Table2.docx]

**Table S2** Key Antibody Information

| **Antibodies** | **Source** | **Identifier and utilization** |
| --- | --- | --- |
| Anti-Src Antibody | HuaBio | #ET1702-03, 1:1000 dilution |
| Anti-Phospho-Src (y419) Antibody | HuaBio | #ET1609-15, 1:1000 dilution |
| AKT Rabbit Poly Antibody | Proteintech | #10176-2-AP, 1:1000 dilution |
| Anti-Phospho-AKT (ser473) Antibody | Nature BioscienceS | #A96286, 1:1000 dilution |
