# Supplementary material for: Association between primary aldosteronism and type 2 diabetes and identification of potential molecular mechanisms
Source: Front Endocrinol (Lausanne). 2026 Aug 4;17:1863900. doi: 10.3389/fendo.2026.1863900 (PMC13481238; doi:10.3389/fendo.2026.1863900)
Supplement: Supplementary file 3 [file Table3.docx]

**Table S3 SNPS in the dataset used for MR Analysis**

| **SNP** | **effect_allele** | **other_allele** | **beta.exposure** | **se.exposure** | **pval.exposure** | **mr_b** | **mr_se** | **mr_p** |
| --- | --- | --- | --- | --- | --- | --- | --- | --- |
| rs284277 | C | A | 0.59 | 0.10 | 1.09E-08 | 0.04 | 0.02 | 0.06 |
| rs17076092 | C | T | -0.52 | 0.11 | 4.87E-06 | 0.02 | 0.02 | 0.41 |
| rs1535532 | C | T | -0.77 | 0.11 | 1.31E-12 | 0.03 | 0.01 | 0.06 |
