# Supplementary material for: Association between primary aldosteronism and type 2 diabetes and identification of potential molecular mechanisms
Source: Front Endocrinol (Lausanne). 2026 Aug 4;17:1863900. doi: 10.3389/fendo.2026.1863900 (PMC13481238; doi:10.3389/fendo.2026.1863900)
Supplement: Supplementary file 4 [file Table4.docx]

**Table S4 SNPS in the dataset used for MR Analysis**

| **SNP** | **effect_allele** | **other_allele** | **beta.exposure** | **se.exposure** | **pval.exposure** | **mr_b** | **mr_se** | **mr_p** |
| --- | --- | --- | --- | --- | --- | --- | --- | --- |
| rs7903146 | T | C | 0.29 | 0.01 | 4.67E-152 | -0.48 | 0.36 | 0.19 |
| rs9273363 | A | C | 0.11 | 0.01 | 1.55E-21 | -0.95 | 1.16 | 0.42 |
| rs13266634 | T | C | -0.11 | 0.01 | 1.23E-20 | 1.00 | 0.99 | 0.31 |
| rs2237895 | C | A | 0.09 | 0.01 | 1.03E-16 | 0.78 | 1.07 | 0.46 |
| rs11708067 | G | A | -0.10 | 0.01 | 1.27E-16 | -0.35 | 1.18 | 0.77 |
| rs1215468 | G | A | -0.09 | 0.01 | 7.31E-15 | 0.97 | 1.16 | 0.40 |
| rs2796441 | A | G | -0.07 | 0.01 | 1.15E-11 | -1.27 | 1.37 | 0.36 |
| rs11651755 | T | C | -0.07 | 0.01 | 2.67E-11 | 1.37 | 1.39 | 0.32 |
| rs1496653 | G | A | -0.08 | 0.01 | 3.36E-10 | 1.58 | 1.44 | 0.27 |
| rs231361 | A | G | 0.07 | 0.01 | 2.20E-09 | 1.25 | 1.58 | 0.43 |
